# Hepatitis C virus (HCV) genotype 1b displays higher genetic variability of hypervariable region 1 (HVR1) than genotype 3

**DOI:** 10.1038/s41598-019-49258-y

**Published:** 2019-09-06

**Authors:** Maciej Janiak, Karol Perlejewski, Piotr Grabarczyk, Dorota Kubicka-Russel, Osvaldo Zagordi, Hanna Berak, Sylwia Osuch, Agnieszka Pawełczyk, Iwona Bukowska-Ośko, Rafał Płoski, Tomasz Laskus, Kamila Caraballo Cortés

**Affiliations:** 1 Department of Immunopathology of Infectious and Parasitic Diseases, Medical University of Warsaw, Warsaw, Poland; 2 Department of Virology, Institute of Hematology and Transfusiology, Warsaw, Poland; 3 Institute of Medical Virology, University of Zurich, Zurich, Switzerland; 4 Outpatient Clinic, Warsaw Hospital for Infectious Diseases, Warsaw, Poland; 5 Department of Medical Genetics, Medical University of Warsaw, Warsaw, Poland; 6 Department of Adult Infectious Diseases, Medical University of Warsaw, Warsaw, Poland

**Keywords:** Hepatitis C virus, Molecular medicine

## Abstract

Hepatitis C virus (HCV) is characterized by high genetic variability, which is manifested both at the inter-host and intra-host levels. However, its role in the clinical course of infection is less obvious. The aim of the present study was to determine the genetic variability of HCV HVR1 (hypervariable region 1) of genotype 1b and 3 in plasma of blood donors in the early seronegative stage of infection (HCV-RNA+, anti-HCV−) and in samples from chronically infected patients using next-generation sequencing. Sequencing errors were corrected, and haplotypes inferred using the ShoRAH software. Genetic diversity parameters (intra-host number of variants, number of nucleotide substitutions and diversity per site) were assessed by DNA SP and MEGA. During the early infection, the number of variants were significantly lower in subjects infected with genotype 3 than with genotype 1b (p < 0.02). Similarly, intra-host number of variants, number of nucleotide substitutions and diversity per site were lower in genotype 3 chronic infection (p < 0.0005). In addition, early infection was characterized by significantly lower HVR1 variability values (p < 0.04) when compared to chronic infection for both genotypes. It seems that the observed differences in HVR1 variability represent an inherent property of particular viral genotypes.

## Introduction

Hepatitis C virus (HCV) is characterized by high genetic variability, resulting from both high error rate of viral RNA polymerase and immune pressure of the infected host. Consequently, HCV intra-host population displays high diversity reflected by the concomitant presence of multiple variants. Typically, after initial reduction upon the virus transmission to a new host, known as transmission bottleneck, HCV diversity expands as patient is progressing to chronicity^1,2^. Genetic variability of intra-host population, driven by immune pressure, allows the virus to escape from both humoral and cellular immune responses, gaining fitness advantage which may result in viral persistence^3,4^. Thus, the composition and dynamics of HCV population during the early phase of infection may determine its outcome^5–8^. HCV inter-patient variability is manifested mainly by the existence of different genotypes, classified from 1 to 7^9^. Despite the fact that they represent the same species, their genome may differ by up to 31–33%^10^. Interestingly, the question whether HCV genotypes are characterized by different intra-host variability remains unresolved. This is partly due to technical limitations of methods utilized to analyze viral diversity, such as DNA heteroduplex gel shift method, single-strand conformation polymorphism or bulk clonal sequencing^11–13^. Their sensitivity for minor variant detection is low, with the variant frequency detection limit no better than 3–15% in most of the studies^1,14^. More recently, novel methods allowed for in-depth analysis of viral diversity such as single-genome or next-generation sequencing (NGS)^2,15^. They provide a tool for evaluation of a wide spectrum of genetic variants, but at a cost of greater sequencing error when compared to clonal sequencing^16^. Concomitantly, novel computational methods aimed at NGS error correction and variants reconstruction have been devised^15,17^.

The aim of the present study was to infer genetic variability of HVR1 (hypervariable region 1), a highly variable fragment of envelope 2 glycoprotein, which is a major target for specific humoral immune response^18^, in an unique collection of plasma samples from blood donors at an early seronegative stage of HCV genotype 1b or 3 infection (HCV-RNA positive, anti-HCV-negative) using a next-generation sequencing strategy. Samples from genotype matched, seropositive chronically infected patients were also analyzed. Our study showed that HCV genotype 3 displays significantly lower HVR1 genetic variability than genotype 1 during both early acute and chronic infection.

## Results

In total, 388 202 HVR1 sequence reads were obtained, 4006.8 (mean per sample) for early infection seronegative subjects and 3595.7 (mean per sample) for chronically-infected patients. Similar number of reads (depth of sequencing) was obtained for genotype 1b and genotype 3 samples, both in early (4060.0 *vs* 3947.2) and chronic infection (3217.3 *vs* 4033.7).

**Figure 1:**
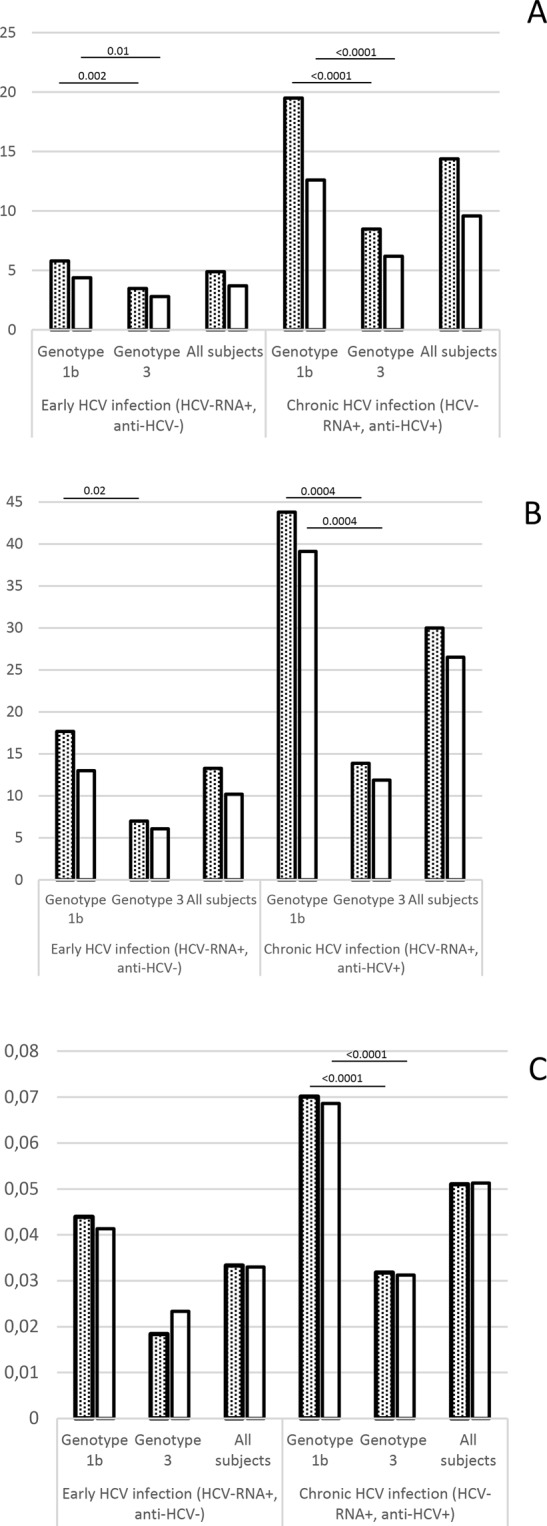
Nucleotide variability parameters of HVR1 HCV variants in genotype 1b and genotype 3 in early and chronic infection after application of 0.5% (dotted bars) and 1% (plain bars) frequency cutoff. (**A**) Number of HVR1 nucleotide variants, (**B**) number of nucleotide substitutions, (**C**) nucleotide diversity per site. The parameters shown in parts (**B**,**C**) were calculated with respect to consensus sequence (the most represented sequence within the sample). Numbers over the horizontal bars represent p-values.

The reads were further corrected for errors and reconstructed into haplotypes using ShoRAH. From one to 31 variants were identified per sample in early infection subjects (three to 31 for genotype 1b and one to 16 for genotype 3) and from five to 114 for chronically-infected patients (five to 114 for genotype 1b and five to 39 for genotype 3). After implementation of 0.5% cutoff to the reconstructed variants, from one to 14 variants were retained per sample in early infection subjects (one to 14 for genotype 1b and one to ten for genotype 3) and from one to 36 in chronically-infected patients (five to 36 for genotype 1b and one to 21 for genotype 3). Consequently, the mean number of nucleotide variants was significantly lower in genotype 3 infected subjects than in those infected with genotype 1b, both for early (3.5 *vs* 5.9; p = 0.002) and chronic (8.5 *vs* 19.5; p < 0.0001) infection (Fig. 1). Likewise, the number of intra-population nucleotide substitutions, when compared to consensus sequence, was significantly lower in case of genotype 3, both for early (7.0 *vs* 17.7 p = 0.02) and chronic (13.9 *vs* 43.8, p = 0.0004) infection. Similarly, the nucleotide diversity per site π was significantly lower in chronic genotype 3 than genotype 1b infection (0.0318 *vs* 0.0701; p < 0.0001). It was also lower in early genotype 3 infection, but this difference did not reach statistical significance (0.0184 *vs* 0.0439; p = 0.053). The summarized results are presented graphically on Fig. 1.

When early and chronic infections were compared, the number of HCV variants, substitutions and the extent of nucleotide diversity were all significantly higher (p < 0.04) in the latter group for both genotype 1b and genotype 3.

In order to verify whether the observed effect is not caused by low frequency erroneous variants, we also re-assessed the variability parameters after increasing the cutoff to 1%. While the number of variants has decreased both for early and chronic infection, the statistical significance of difference between the two genotypes was still retained (p = 0.01 for early and p < 0.0001 for chronic infection, Fig. 1). Likewise, with the application of 1% cutoff, the number of substitutions and the extent of nucleotide diversity were still significantly lower in chronic genotype 3 compared to chronic genotype 1b infection (p = 0.0004 and p < 0.0001, respectively). In case of early genotype 3 infection these parameters were also lower, but not statistically significant (p = 0.08 and p = 0.16, respectively).

**Figure 2:**
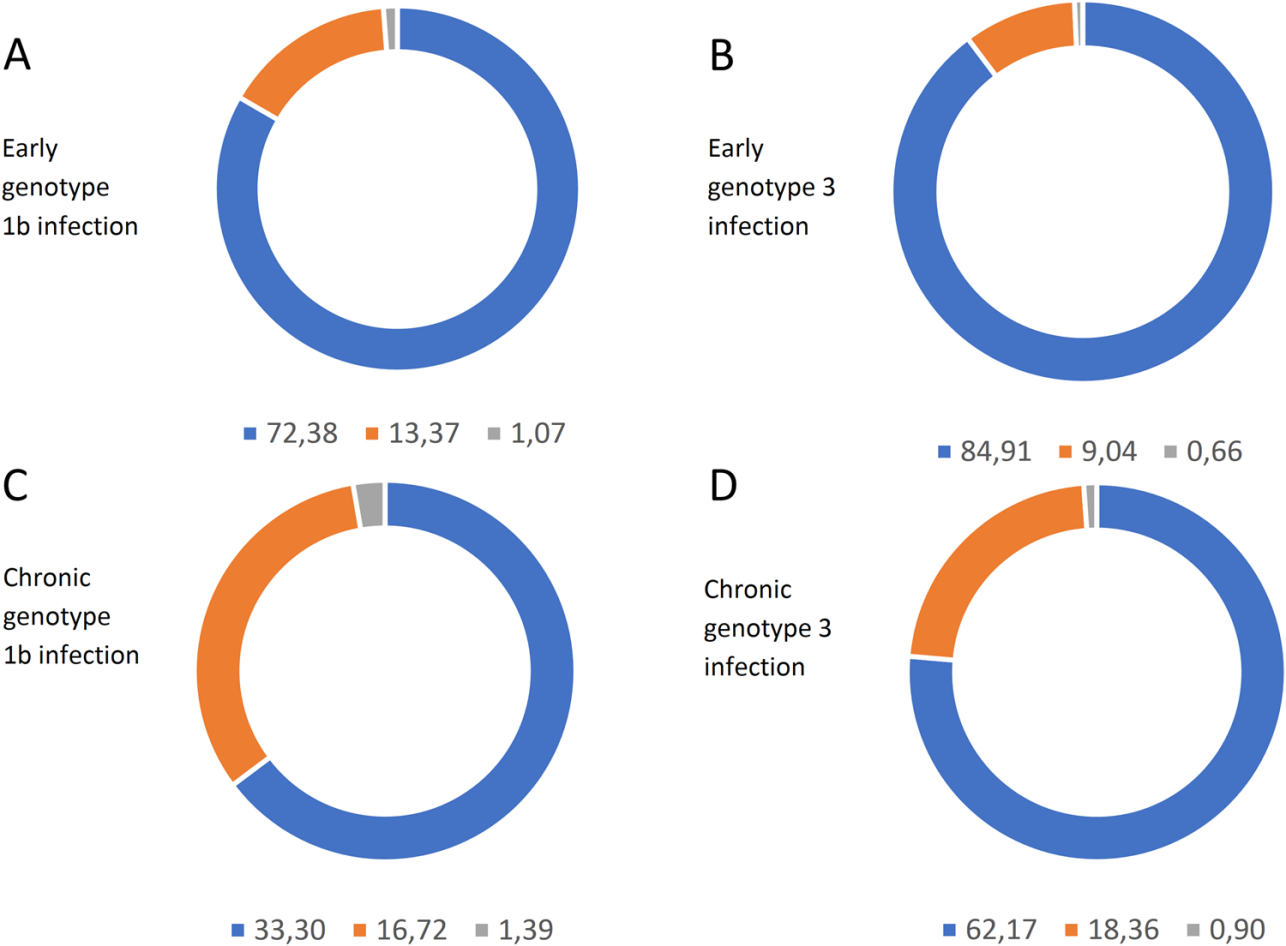
Mean frequencies of two most prevalent HCV HVR1 variants in early infection with genotype 1b (**A**) and genotype 3 (**B**) and in chronic infection with genotype 1b (**C**) and genotype 3 (**D**). The frequency of each variant was determined by LStructure from ShoRAH suite and ranked from highest to lowest. Next, mean values were calculated for each of the two highest frequency variants in each patient (shown in blue and orange) and for the remaining ‘other’ variants (shown in gray). The applied frequency cutoff was 0.5%.

Comparison of intra-host populations revealed that genotype 3 HVR1 displayed higher frequency contribution of the dominant (i.e. the most frequent) variant than genotype 1b (mean 84.9% *vs* 72.4% for early infection, p = 0.004 and 62.2% *vs* 33.3% for chronic infection, p = 0.0003). The frequency distribution of major variants in genotype 1b and 3 infection using 0.5% cutoff is presented on Fig. 2.

## Discussion

Our study aimed to characterize in-depth intra-host genetic variability of HCV genotypes 1b and 3, both at the very early phase of infection prior to seroconversion, reflecting the natural complexity of the virus in the absence of humoral pressure and in a group of seropositive, treatment-naïve chronically infected patients. A unique opportunity to study very early virus-related events was provided by access to samples from HCV RNA positive, anti-HCV negative donors. Such samples represent very early infection stage and are detected at a frequency of 18.5 per 1 million donations^19^.

We found that during the early infection, the number of HVR1 variants was significantly lower in subjects infected with genotype 3 than in those infected with genotype 1b. Importantly, the effect observed was neither related to number of reads nor to viral titer. Furthermore, the results of analysis at two different cutoffs (0.5% and 1%) were concordant. The detected differences between HCV genotype 1b and 3 HVR1 molecular characteristics are striking and to our best knowledge no previous study addressed this issue.

To determine whether differences in HVR1 variability are affected by serologic response, a similar comparison was performed in a group of seropositive, chronically-infected patients. Since differences between the two genotypes were still maintained, it seems that genotype 3 HVR1 displays lower evolutionary dynamics than genotype 1b during the entire course of infection. Nevertheless, HVR1 variability parameters were significantly higher in chronic infection compared to early infection, which is concordant with earlier findings of bottleneck effect, due to selective transmission of only some HCV variants^2,8,13^. Subsequently, gradual expansion of the intra-host population occurs, which can be eventually disturbed by environmental pressures or fitness costs to the virus^1,20^.

Importantly, both during early and chronic genotype 3 infection, there was a different intra-host frequency structure of variants than during genotype 1b infection, with higher frequency contribution of a major (i.e. the most frequent) variant. This implies that genotype 3 has lower capacity to form minor frequency variants, which could provide fitness advantage under environmental pressures, such as exerted by the immune system or treatment. It can be speculated that this is the reason why genotype 3 infection is characterized by better response to immunomodulatory treatment with interferon alpha and ribavirin^21^. The differences in HVR1 genetic variability may also have clinical implications for the natural course of infection. Some observations imply that while genotype 3 is responsible for the majority of community-acquired HCV infections, is often eliminated at the early stage of infection and its potential to establish chronic infection is lower when compared to other HCV genotypes, particularly 1b^19,22^. In a study of Hwang *et al*., comprising 67 patients with acute post-transfusion hepatitis C, patients with genotype 1b were more likely to develop chronic hepatitis than patients infected with other genotypes (89.7% vs. 64.3%; p = 0.019)^23^. Similarly, Amoroso *et al*. showed, that the rate of progression to chronicity was 92% in patients exposed to HCV genotype 1b compared to 50% in patients exposed to genotype 3^6^. These data provide evidence that HCV genotype may play an important role in the clinical course of infection following exposure to HCV. Lower genetic variability of genotype 3 observed by us during the seronegative early phase of infection may be related to higher chance of spontaneous elimination of the virus, since the immune system may be more efficient at targeting “uniform” viral population^3^.

While NGS is an extremely useful tool for the evaluation of genetic diversity, it is highly prone to sequencing errors. Because of that, we conducted an extensive assessment of the inherent raw sequencing error of the whole procedure using HVR1 plasmid insert as template^24^, applied subsequent error correction and frequency cutoff. Importantly, the differences were observed at two different cutoffs (i.e. 0.5% and 1%).

It should be noted that, while we found evident differences between the genotype 1b and genotype 3 variability, these observations are limited to immunologically important, yet single and small genomic region and may not extend to other hypervariable regions of HCV such as HVR2 and HVR3^25^. Notably, novel hypervariable regions HVR495 and HVR575 have been found to be present in genotype 3, but not in genotype 1a, 1b, 2a or 6a genomes^26^. Since these are also subject to positive immune selection, further research is needed to determine their variability characteristics in early and chronic infection.

In conclusion, the HVR1 genetic variability parameters were lower in patients infected with genotype 3 than in patients infected with genotype 1b. Considering that these differences could be observed both in acute and chronic infection, it seems that the phenomenon is not due to immune pressure, but may rather represent an inherent property of particular viral genotypes.

## Methods

### Patients

The study encompassed plasma samples from 60 blood donors who were found to be HCV RNA positive and anti-HCV negative at the time of blood donation. Since their samples collected 3–6 months earlier were negative for the presence of HCV RNA and anti-HCV, they were assumed to be recently infected. Importantly, the time from the last blood donation, in which HCV-RNA and anti-HCV were negative, was identical for genotype 1b and genotype 3-infected donors (median 3 months, p = 0.838). These samples have been collected in the years 1999–2013 as part of a routine blood donor screening conducted by Polish blood donation centers and the Institute of Hematology.

Plasma samples from 41 HCV RNA–positive, anti-HCV-positive treatment-naïve chronic hepatitis C patients infected with genotype 1b or 3, presenting for treatment at the Hepatology Outpatient Clinic of the Warsaw Hospital for Infectious Diseases were also analyzed. Although the exact duration of infection was not known, the time from the initial diagnosis of HCV infection was similar (mean 3.9 years for genotype 1b *vs* 3.0 years for genotype 3).

The study protocol followed ethical guidelines of the 2013 Declaration of Helsinki and was approved by the Bioethical Committee of the Medical University of Warsaw (Approval Number KB/17/2013 and KB/107/2010). All patients provided written informed consent. Some clinical and virological characteristics of the study subjects are presented in Table 1.

**Table 1:** Clinical and virological characteristics of studied subjects.

	HCV-RNA-positive, anti-HCV-negative blood donors				Chronically HCV-infected patients			
	HCV Genotype 1 (n = 35)	HCV Genotype 3 (n = 25)	P	All patients (n = 60)	HCV Genotype 1 (n = 22)	HCV Genotype 3 (n = 19)	P	All patients (n = 41)
Subtype	1b n = 35	3a n = 24 Undetermined subtype n = 1	N/A		1b n = 22	3a n = 18 Undetermined subtype n = 1	N/A	
Sex [M/F]	33/2	20/5	NS	53/7	10/12	11/8	NS	21/20
Age (years, mean [median], range)	28.3 [25.0]; 18–55	26.2 [23.0]; 19–49	NS	27.4 [25]; 18–55	45.1 [50.0]; 19–68	38.5 [39.0]; 24–58	NS	42.1 [40]; 19–68
Alanine aminotransferase levels [U L^−1^]; mean [median], range) ref. values: 10–40 U L^−1^	50.0 [47.0]; 9–143	39.8 [37]; 6–74	NS	46.1 [44]	94.1 [73.5] 25–246	157.6 [133]; 36–400	0.03	120.8 [80]; 25–400
Viral load [IU mL^−1^]^b^; mean [median], range)	4 700 257 [793 000]; 184–47 800 000	5 253 725 [603500]; 2650–69 000 000	NS	4 907 808 [775 500]; 184–69 000 000	1 219 045 [846 000.0]; 102 000–3 980 000	2 642 312 [1 305 000]; 246 000–19 900 000	NS	1 818 315 [1 007 500]; 102 000–19 900 000

^b^Assessed using Cobas Amplicor HCV Monitor test v 2.0 (Roche Molecular Systems, South Branchburg, USA).

N/A-not applicable.

NS-not statistically significant.

### HVR1 amplification

HVR1 amplification was performed as described previously^27^. In brief, total RNA was extracted from 250 μl of plasma by a modified guanidinium thiocyanate-phenol/chlorophorm method using Trizol (Life Technologies, Carlsbad, CA, USA). RNA was subjected to reverse transcription at 37 °C for 30 minutes using AccuScript High Fidelity Reverse Transcriptase (Agilent Technologies, Santa Clara, CA, USA) and random hexamers (Life Technologies). A gene fragment encompassing HVR1 was amplified in two-step PCR using FastStart High Fidelity Taq DNA Polymerase (Roche, Indianapolis, IN, USA) using target-specific primers (Table 2). Additionally, primers employed in the second round PCR contained tags recognized by GS Junior sequencing platform and standard 10-nucleotide multiplex identifiers^27^.

**Table 2:** Primer sequences employed in the study.

	genotype 1b HVR1 amplification	Positions of HCV genome	genotype 3 HVR1 amplification	Positions of HCV genome
First round PCR	Forward: 5′-GGTGCTCACTGGGGAGTCCT-3′	1389–1408	Forward: 5′-ATGGCATGGGATATGAT-3′	1291–1307
	Reverse: 5′-CATTGCAGTTCAGGGCCGTGCTA-3′	1632–1610	Reverse: 5′-AAGGCCGTCCTGTTGA-3′	1619–1604
Second round PCR	Forward: 5′- TCCATGGTGGGGAACTGGGC-3′	1428–1447	Forward: 5′-GGCAACTGGGCCAAGGTCGC-3′	1437–1456
	Reverse: 5′-TGCCAACTGCCATTGGTGTT-3′	1603–1584	Reverse: 5′-ATGTGCCACGAGCCATTGGT-3′	1606–1587

### Pyrosequencing

The amount of DNA equivalent to 3 × 10^7^ amplicons was subjected to emulsion PCR using GS Junior Titanium emPCR Lib-A Kit (454 Life Sciences, Branford, CT, USA). Pyrosequencing was carried out according to the manufacturer’s protocol for amplicons using GS Junior System (454 Life Sciences).

### Data analysis

Sequencing errors (mismatches, insertions and deletions) were corrected and haplotypes inferred using the program diri_sampler from the ShoRAH software (https://www1.ethz.ch/bsse/cbg/software/shorah)^17^. It employs a local reconstruction model based on probabilistic clustering algorithm to correct errors^28^. This is accomplished by clustering all reads that overlap the same region of the genome of length approximately equal to the read length. The consensus sequence of each cluster represents the original haplotype from which the erroneous reads are obtained. ShoRAH does not feature a generative model for indels correction, but rather relies on the sequence alignment. Each read is aligned to the reference, and from this set of pairwise alignments a multiple one is built. If a given indel is present in a sufficient number of reads (i.e. if the model identifies it as real variation and not as a sequencing error), it will be present in one or more haplotypes.

The raw error rate of amplification and pyrosequencing of a clone-derived amplicon was shown in our previous study to be 1.5% (measured as the frequency of the most prevalent erroneous variant)^24^. However, error correction by ShoRAH reduced this error to 0.5% and this cutoff was thus implemented in our analysis. Nevertheless, in order to verify our results, the analysis was repeated using 1% cutoff.

Haplotypes that had posterior probability >95% and were represented by at least 10 reads were extracted with LStructure (https://github.com/ozagordi/LocalVariants/blob/master/src/LStructure.py). Subsequently, haplotypes were aligned to the HCV reference sequences (GenBank accession number AJ406073 for genotype 1b and EF442261.1 for genotype 3) and trimmed to be equal in length^29^. Genetic diversity parameters (i.e. intra-host number of variants, number of nucleotide substitutions and nucleotide diversity per site π) were assessed by DNA SP version 5 (http://www.ub.edu/dnasp/)^30^ and MEGA 5.0^29^.

### Statistical analysis

Significance of differences in HVR1 sequence diversity and clinical data (ALT levels, viral load) were tested using the Unpaired T test or Mann-Whitney U test where appropriate based on the Kolmogorov-Smirnov normality test. Differences in sex distribution between HCV genotypes were tested using Fisher’s exact test. All p-values were two tailed and considered significant when <0.05.

## Data Availability

All the HVR1 sequences are publically available at the Zenodo repository (10.5281/zenodo.3266795).
